# Human Cytomegalovirus variant peptides adapt by decreasing their total coordination upon binding to a T cell receptor

**DOI:** 10.1016/j.dib.2015.07.019

**Published:** 2015-07-26

**Authors:** Georgios S.E. Antipas, Anastasios E. Germenis

**Affiliations:** a Division of Materials Technology, National Technical University of Athens, Zografou Campus, Athens 15780, Greece; b Department of Immunology & Histocompatibility, School of Medicine, University of Thessaly, Biopolis, Larissa 41110, Greece

**Keywords:** pMHC-TCR interactions, Class I MHC, CD8^+^ Cytotoxic lymphocytes, Protein–protein interactions

## Abstract

The tertiary structure of the native Cytomegalovirus peptide (NLV) presented by HLA-A2 and bound to the RA14 T cell receptor was used as a reference for the calculation of atomic coordination differences of both the NLV as well as of a number of singly substituted NLV variants in the absence of TCR. Among the pMHC complexes, the native peptide was found to exhibit the highest total coordination difference in respect to the reference structure, suggesting that it experienced the widest structural adaptation upon recognition by the TCR. In addition, the peptide on the isolated NLV-MHC complex was over-coordinated as compared to the rest of the variants. Moreover, the trend was found to account for a set of measured dissociation constants and critical concentrations for target-cell lysis for all variants in complexation with RA14: functionally, all variant peptides were established to be either weak agonists or null peptides, while, at the same time, our current study established that they were also under-coordinated in respect to NLV. It could, thus, be argued that the most ‘efficient’ structural adaptation upon pMHC recognition by the TCR requires of the peptide to undergo the widest under-coordination possible. The main structural characteristic which differentiated the NLV in respect to the variants was a the presence of 16 oxygen atoms (waters) in the former׳s second coordination shell which accounted for over-coordination of roughly 100% and 30% in the O–O and C–O partials respectively. In fact, in the absence of second shell oxygens, the NLV peptide was decidedly under-coordinated in respect to all of the variants, as also suggested by the C–C partial.

**Table:** Specifications table

Subject area	Immunology, biochemistry, materials science, quantum chemistry
More specific subject area	Class I MHC, CD8^+^ Cytotoxic lymphocytes, protein–protein interactions
Type of data	Excel spreadsheet
How data was acquired	Data from crystallized tertiary structures was acquired from the Protein Data Bank (PDB)
Data format	Text
Experimental factors	None
Experimental features	None
Data source location	Not applicable
Data accessibility	Data is with this article

Value of the data

• On the basis of our previous findings which suggest that peptide-Major Histocompatibility Complex (pMHC) binding affinity with a T Cell Receptor (TCR) may be inferred from peptide tertiary structure [1], [2], our current results highlight the possible inference of peptide immunological identity from its tertiary structure on the MHC, in the absence of complexation to a TCR. Peptides on the isolated pMHC complexes were substantially over-coordinated in respect to the (native) NLV-A2-RA14 complex. Over-coordination was owing to the oxygen partials.
• As shown in Fig. 3a, the native peptide appears to be able to undergo the widest possible under-coordination when recognized by a TCR.

## 1 Introduction

Several lines of evidence indicate that Cytomegalovirus (CMV), a ubiquitous *β*-herpesvirus that infects 60–90% of the population, is a driving force in age-related T cell immunosenescence. Briefly, in CMV-seropositive older adults, aging has been associated with large expansion of CMV-specific CD8^+^ T cell clones and shrinkage of the T cell repertoire available for other antigens [3], [4], [5], [6], [7]. In individuals sharing the widespread HLA-A*0201 allele (referred to as A2), CMV-specific CD8^+^ T cells recognize the same epitope pp65_495–503_ (NLVPMVATV), hereafter referred to as NLV [8], [9].

Despite the great effort made to date, the founding mechanism of NLV immunodominance has not been resolved. However, studies thus far have indicated that antigen-driven selection is most likely the main parameter contributing to the predominant usage of public TCR by NLV-A2-specific CD8 T cells [10], [11], [12]. Motivated by our previously reported finding that pMHC-TCR binding avidity of the transcriptional regulatory protein of the human T-cell leukemia virus type 1 (HTLV-1), Tax, along with that of a number of its variants with A6 TCR was highly correlated to atomic coordination of peptide tertiary structure [1], [2], here we investigate the potential correlation between atomic coordination of a number of NLV variant-MHC complexes in respect to that of the NLV-MHC and we additionally inquire whether this correlation may also be indicative of the end effect of pMHC-TCR binding avidity of the same complexes as recognized by the RA14 TCR.

## 2 Materials and methods

### 2.1 Peptides

The current study considered a number of Cytomegalovirus (CMV) peptide (NLV) variants, all of which are presented by HLA-A2. Each variant was synthesized via a single residue substitution on the NLV [11]. In our work, the tertiary structures of the variants were compared to that of the native peptide (3GSN_P), the tertiary structure of which was isolated from its complex with HLA-A2 bound to the RA14 T TCR (PDB accession code 3GSN). The pMHC complexes studied are listed in Table 1.

### 2.2 Calculation of pair correlation functions

For all peptides in Table 1, as well as for the native peptide, the Pair Distribution Function (PDF, symbolized as *g*(*r*)) was calculated with a bin size equal to 0.1 Å, by

(1) $$g(r)=\frac{1}{2\pi Nr^{2}\rho_{0}}\sum_{j=1}^{N}\sum_{i>j}^{N}\delta (r-r_{ij})$$

where *N* is the number of peptide atoms, *δ* is the Dirac delta function and *ρ*_0_ is the number density *N*/*V*, *V* is the volume of the simulation box containing the peptide. In (1), if the species of the *i*th and/or *j*th are restricted, the *g*(*r*) calculated represents a partial (e.g. if all *i* atoms are restricted to C the PDFs computed would be the carbon partials; additionally if the *j* atoms are also restricted to, e.g., oxygen, the partial computed would be the C–O), otherwise it is the total PDF. The Radial Distribution Function (RDF, symbolized as *R*(*r*)), was then calculated as

(2) $$R\left(r\right)=4\pi r^{2}\rho_{0}g\left(r\right)$$

and integrated to estimate the cumulative atomic coordination, *n*^*r*1^, of any atom within a sphere of radius *r*_1_ as follows

(3) $$n^{r_{1}}=\int_{0}^{r_{1}}R(r)dr=4\pi \rho_{0}\int_{0}^{r_{1}}g(r)r^{2}dr$$

**Fig. 1:**
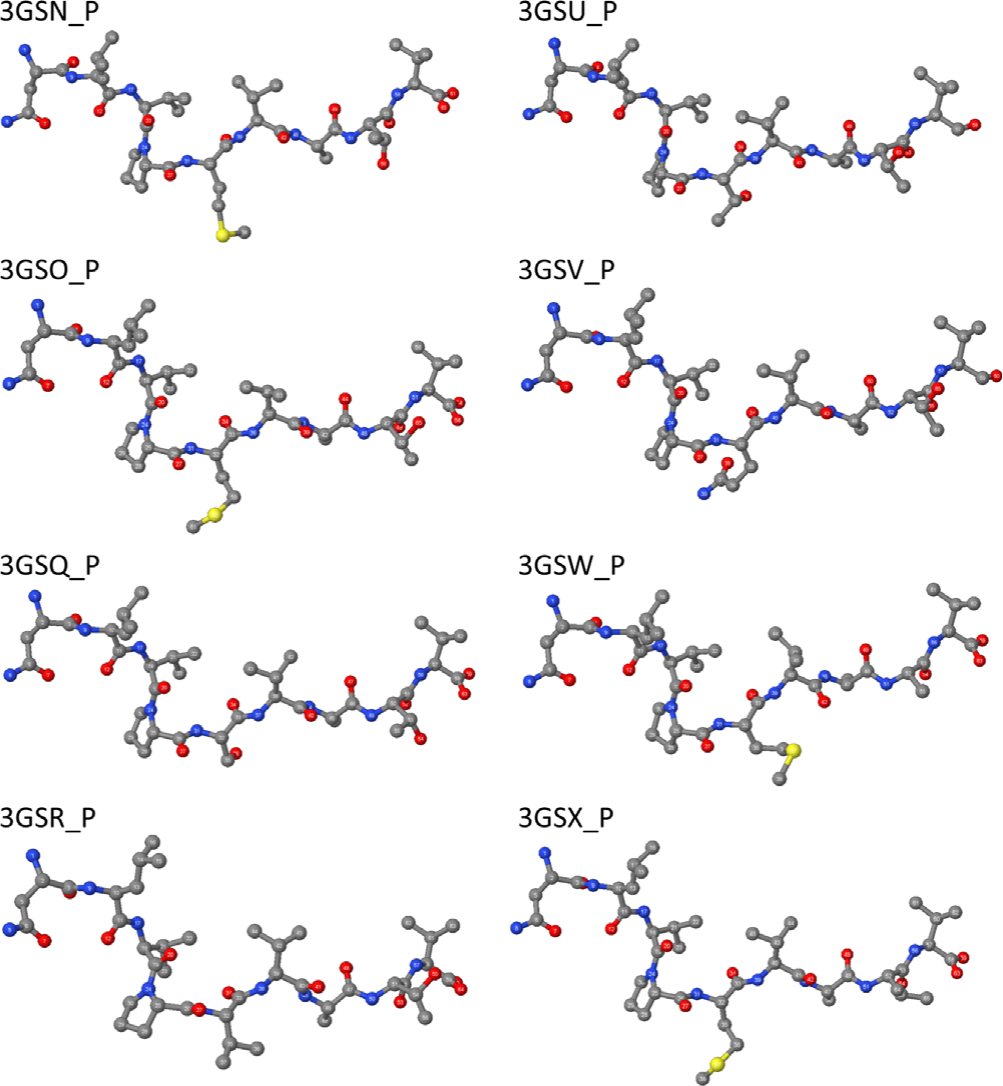
The stereochemistries of an indicative subset of peptides considered in the current study. In all peptides shown, second coordination shell oxygen atoms have been excluded. The peptides are (also see model designation in Table 2): A1 (3GSO_P), A4 (3GSQ_P), A5 (3GSR_P), A7 (3GSU_P), A10 (3GSV_P), A13 (3GSW_P) and A14 (3GSX_P). Reference peptide 3GSN_P is also shown.

Hence the running difference between the coordination of the peptide on the NLV-A2-RA14 complex and each of the peptides in Table 1 was calculated. These running differences in cumulative coordination are presented in Fig. 1. It follows from the definition of the PDF (Eq. 1) that both total and partial RDF and coordination may be calculated. All calculations of PDF, RDF and coordination were performed with the PRDF program [13], [14], [15], [16].

### 2.3 Side chain mobility and Density Functional Theory calculations

In the case of the 3GSO_P, 3GSV_P, 3GSU_P and 3GSX_P complexes increased side-chain mobility resulted in multiple atomic conformations as recorded via X ray diffraction in the work by Gras et al. [11]. Here, for the calculation of the pair correlation functions we created unique structural models (see ‘Number of different pMHC models’ in Table 1) for each peptide with multiple atom positions via the following two separate routes:

a) If an atom׳s multiple positions differed by less than its covalent radius, all positions but one were discarded as small PDF histogram differences do not contribute appreciably to the total coordination of the peptide. In cases where an atom׳s possible positions were separated by more than the covalent radius, we produced different peptide models having both included and excluded second coordination shell oxygen atoms and calculated coordination for each model. All models studies are listed in Table 2.
b) To further account for the structural complexity due to side-chain mobility in the 3GSO_P peptide, all of its atoms were first saturated with hydrogens assuming a neutral pH (peptide N and C termini involved NH_3_^+^ and COO^−^ groups, respectively). The side-chain atoms in question along with the entire H species were then allowed to relax as charge-neutral zwitterions via closed-shell Density Functional Theory (DFT), while keeping the rest of the peptide atoms immobile in their crystallographed positions. The DFT relaxed model for 3GSO_P is designated as ‘A3’ in Table 2.

DFT calculations were performed with the Amsterdam density functional (ADF) program [17], [18]. Electron exchange and correlation were addressed within the generalized gradient approximation (GGA) by the BLYP [19], [20] functional. Single-electron wavefunctions were expanded using the TZ2P uncontracted Slater-type orbital (STO) basis set, (a triple-ζ basis set with two sets of polarization functions) for all atoms. Calculations were all-electron for the H species, while for C, N and O core electrons were frozen inclusive of the 1s shell whereas for S the frozen core included 2p. During relaxation, aufbau occupations were always imposed. Self-consistent field (SCF) convergence invariably required use of the Augmented Roothaan-Hall Direct Inversion Iterative Subspace (ADIIS) scheme [21].

## 3 Brief discussion

**Fig. 2:**
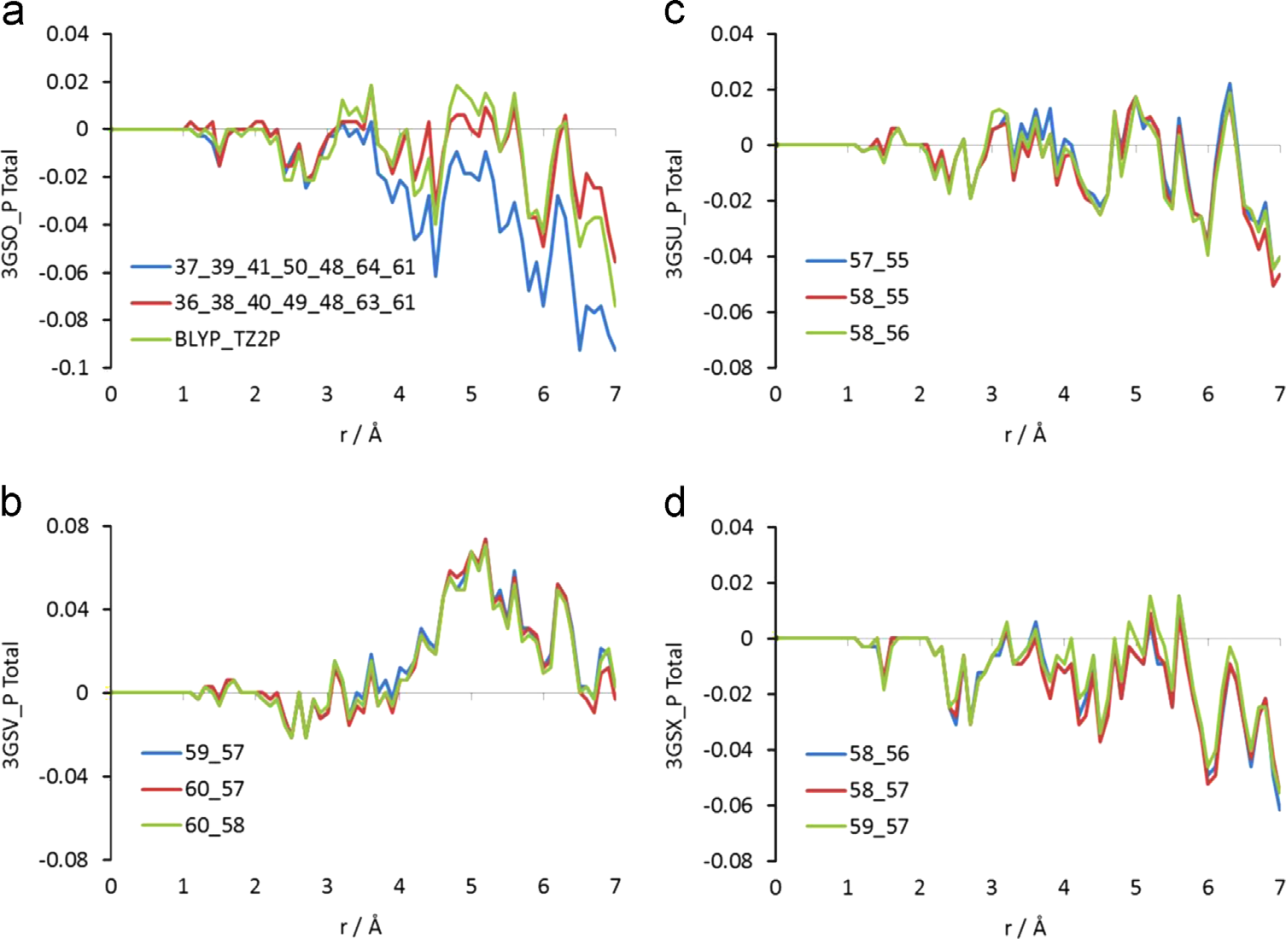
Evolution of the difference between the cumulative total coordination for each of the variants which exhibited increased side-chain mobility, in respect to the native peptide. Coordination has been calculated for unprotonated structures excluding second shell O atoms. (a) Of the conformations possible for 3GSO_P, the two models which yielded the highest and lowest coordination in respect to the native peptide are shown. Additionally, coordination of the model for which side chain atoms were allowed to undergo DFT relaxation is included. (b), (c) and (d) Coordination differences of the three possible models for each of the 3GSV_P, 3GSU_P and 3GSX_P peptides respectively. In the graphs, the data series designation involves the indexes (as they appear in the original PDB entry) of the mobile side-chain atoms present in each model.

**Fig. 3:**
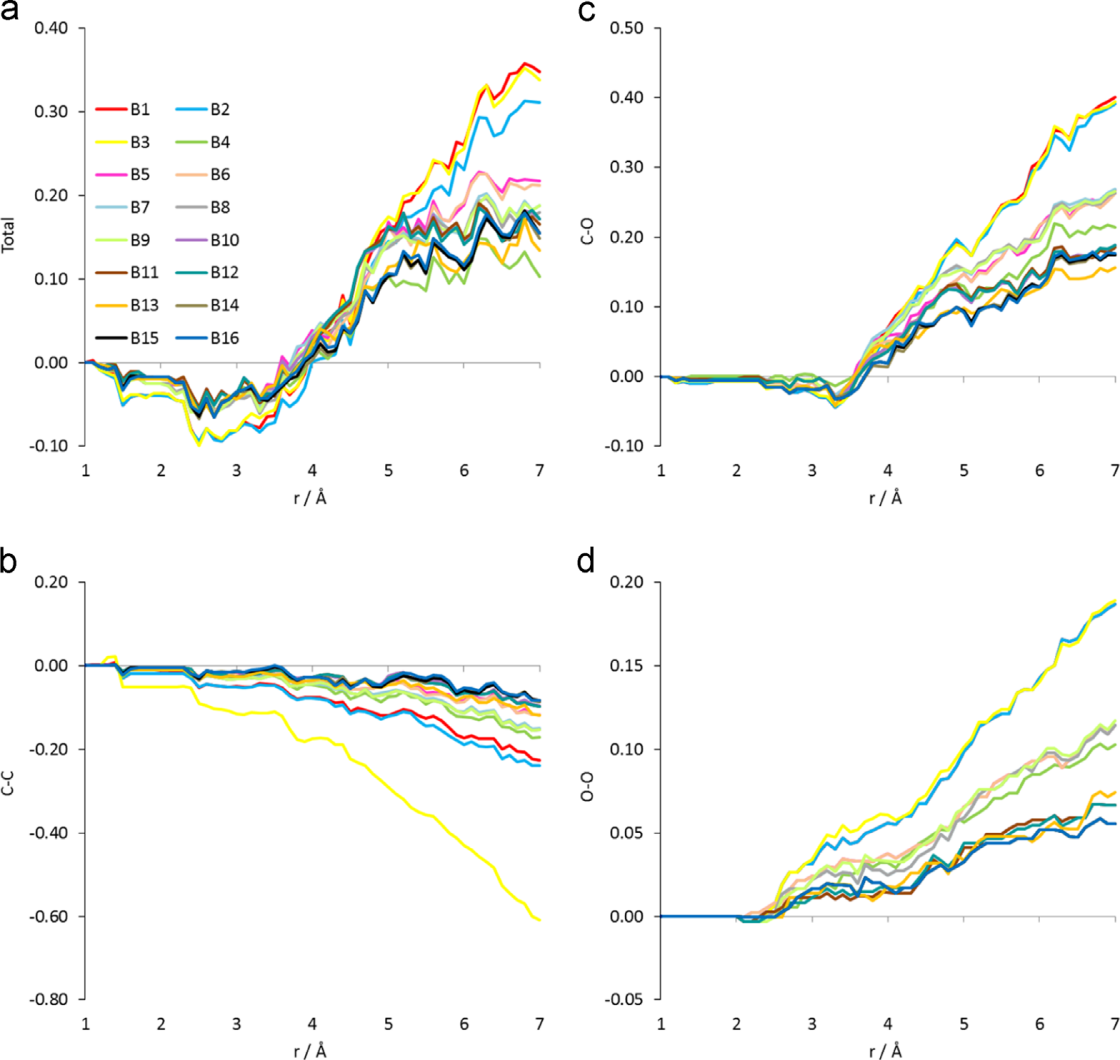
Total and partial cumulative coordination differences between the unprotonated tertiary structures of each variant (Table 1) and the tertiary structure of the peptide on NLV-A2-RA14 complex (PDB ID 3GSN). All variants include second shell oxygen atoms. (a) Total coordination and coordination partials (b) C–C, (c) C–O and (d) O–O.

Indicative peptide stereochemistries are shown in Fig. 1. In the absence of second shell O atoms, the native peptide, 3GSO_P, is under-coordinated in respect to the reference (also see Fig. 3b) and may not be differentiated from the other peptides exhibiting increased side-chain mobility (3GSU_P, 3GSV_P and 3GSX_P in Fig. 1). Additionally, as shown in Fig. 2, the variance of the total coordination of the 3GSO_P models varies is substantially higher, roughly four times that of the other three peptides associated with mobile side-chains. Interestingly, the coordination difference of the DFT-relaxed 3GSO_P model from the reference falls well within the range of 3GSO_P models. It might, thus, be envisaged that the ‘flexibility’ shown by 3GSO_P to adapt to recognition by the TCR rests on the substantially higher number of second shell O atoms – almost twice as many O atoms in comparison to the rest of the variants, also see Table 1. Due to their abundance, second shell O atoms, which represent water molecules coordinated around the peptide might provide adequate ‘cushioning’ to the NLV such that the peptide is able to adjust to a wide variety of TCR׳s via expulsion of waters during recognition [22].

Certainly, inclusion of the second shell O atoms in peptide structures produced a trend of total coordination vs. functional avidity of the peptide in complexation with the TCR which was reminiscent of our results on atomic coordination of pMHC-TCR complexes in the case of Tax and a number of Tax-derived variants [1], [2], i.e. the agonist peptide is over-coordinated in respect to the less agonist/antagonist. As depicted in Fig. 3a, all three 3GSO_P models were over-coordinated in respect to the reference as well as compared to the rest of the variants. In fact, 3GSO_P over-coordination in respect to the variants was almost exclusively due to the C–O and, principally, the O–O partials by roughly 60% (see Fig. 3c and d respectively) as compared to the highest-coordinated of the variants.

## 4 Note on the data files

All peptide structures considered in the current study have been included in .xyz format in the Structures.rar file. The pair correlation data underlying to this work are included in PRDF.rar in comma delimited format. Data comparison is provided in PRDF.xls, which comprises total and partial PDF, RDF and coordination (RDF(*r*)d*r*) data for each of the peptides studied, in respect to interatomic distance, *r* (Å). In each of the tabs, the first line of every column represents the peptide number density (atoms/Å^3^) while the second line is the peptide designation, corresponding to Table 1. The sequence of peptides is the same in each tab. In the set of tabs named ‘PDF XXX’, the peptide data have been calculated via Eq. (1), while data in the tabs named ‘RDF XXX’, the peptide data have been calculated via Eq. (2). The RDF(*r*)d*r* XXX have been calculated via Eq. (3). and to estimate the cumulative coordination for a peptide structure, the running sum of RDF(*r*)d*r* may be calculated as shown in the example tab ‘RDF(*r*)d*r* Total’ and coordination differences in respect to the column named ‘Reference’ may then be calculated also as exemplified.

## Figures and Tables

**Table 1:** All peptides were loaded on HLA-A*0201.

**PDB ID**	**Peptide designation**	**Peptide sequence**	**Number of 2**nd **shell O**	**Number of different pMHC models**	***K***_**D**_	**EC**_**50**_
3GSO	3GSO_P	NLVPMVATV	16	96	27.7	5.10^−2^ ±0.01
3GSV	3GSV_P	NLVPQVATV	6	3	57.5	10^2^±20
3GSQ	3GSQ_P	NLVPSVATV	7	–	n.b.	5.10^2^±20
3GSR	3GSR_P	NLVPVVATV	9	2	60.2	5.10^2^±50
3GSU	3GSU_P	NLVPTVATV	9	3	58.2	10^3^±30
3GSW	3GSW_P	NLVPMVAAV	7	–	n.b.	>10^4^
3GSX	3GSX_P	NLVPMVAVV	6	3	n.b.	>10^4^

All PDB entries listed refer to pMHC complexes. For each of the variants the amino acid substituted on the original NLV sequence (i.e. 3GSO_P) is underlined. Each peptide was surrounded by second coordination shell oxygen atoms (they are hence designated as ‘2nd shell O’ in the table). The number of different models arising for each peptide, due to substantial side-chain mobility during crystallization [11], is listed in the fifth table column. For 3GSO_P multiple positions involved the following atoms (notation is the atom species followed by its index in the PDB entry, multiple positions for the same species are separated by ‘/’): C36/C37, S38/S39, C40/C41 on residue 5, C47/C48/C49/C50 on residue 6, C63/C64 on residue 8 and O61/O62 on residue 8. 3GSV_P and 3GSV_P involved C57/C58 and O59/O60 on residue 8. Gras et al. [11] also measured the functional avidity of each of the variant pMHC complexes upon recognition by RA14 TCR (these pMHC-TCR structures were not crystallographed) and these values are presented as dissociation constants (*K*_D_) and peptide concentration necessary to achieve half maximal target-cell lysis (EC_50_) (notation ‘n.b.’ signifies that no pMHC-TCR binding was detected).

**Table 2:** Peptide models used in the coordination calculations.

Peptide designation	Model	2nd Shell O	
		Excluded	Included
3GSO_P	36_38_40_49_48_63_61	A1	B1
“	37_39_41_50_48_64_61	A2	B2
“	BLYP_TZ2P	A3	B3
3GSQ_P	–	A4	B4
3GSR_P	56	A5	B5
“	57	A6	B6
3GSU_P	57_55	A7	B7
“	58_55	A8	B8
“	58_56	A9	B9
3GSV_P	59_57	A10	B10
“	60_57	A11	B11
“	60_58	A12	B12
3GSW_P	–	A13	B13
3GSX_P	58_56	A14	B14
“	58_57	A15	B15
“	59_57	A16	B16

All models refer to peptide tertiary structure isolated from the pMHC complexes. Peptide designation follows from Table 1 and Model refers to the details given in the caption of Table 1. Model designation starts with ‘A’ and ‘B’ if second coordination shell oxygen atoms were included in and excluded from the structure, respectively.

## Footnotes

Appendix A Supplementary data associated with this article can be found in the online version at doi:10.1016/j.dib.2015.07.019.
